# Potential Signals of Natural Selection in the Top Risk Loci for Coronary Artery Disease: 9p21 and 10q11

**DOI:** 10.1371/journal.pone.0134840

**Published:** 2015-08-07

**Authors:** Daniela Zanetti, Robert Carreras-Torres, Esther Esteban, Marc Via, Pedro Moral

**Affiliations:** 1 Department of Animal Biology-Anthropology, University of Barcelona, Barcelona, Spain; 2 Department of Psychiatry and Clinical Psychobiology and Institute for Brain, Cognition and Behavior (IR3C), University of Barcelona, Barcelona, Spain; 3 Biodiversity Research Institute, University of Barcelona, Spain; IPATIMUP (Institute of Molecular Pathology and Immunology of the University of Porto), PORTUGAL

## Abstract

**Background:**

Coronary artery disease (CAD) is a complex disease and the leading cause of death in the world. Populations of different ancestry do not always share the same risk markers. Natural selective processes may be the cause of some of the population differences detected for specific risk mutations.

**Objective:**

In this study, 384 single nucleotide polymorphisms (SNPs) located in four genomic regions associated with CAD (1p13, 1q41, 9p21 and 10q11) are analysed in a set of 19 populations from Europe, Middle East and North Africa and also in Asian and African samples from the 1000 Genomes Project. The aim of this survey is to explore for the first time whether the genetic variability in these genomic regions is better explained by demography or by natural selection.

**Results:**

The results indicate significant differences in the structure of genetic variation and in the LD patterns among populations that probably explain the population disparities found in markers of susceptibility to CAD.

**Conclusions:**

The results are consistent with potential signature of positive selection in the 9p21 region and of balancing selection in the 9p21 and 10q11. Specifically, in Europe three CAD risk markers in the 9p21 region (rs9632884, rs1537371 and rs1333042) show consistent signals of positive selection. The results of this study are consistent with a potential selective role of CAD in the configuration of genetic diversity in current human populations.

## Introduction

Coronary artery disease (CAD) is a complex disease and the main cause of death in the world. In 2007, the first genome-wide significant risk region, 9p21, was simultaneously discovered by two independent groups [1, 2] and was subsequently confirmed by multiple investigators around the world. Currently, there are a total of 50 variants predisposing to CAD of genome-wide significance confirmed in independent populations [3]. Out of these 50 risk loci, 15 are associated with conventional risk factors for CAD: seven with low-density lipoprotein-cholesterol (LDL-C); one with high-density lipoprotein (HDL); two with triglycerides; four with hypertension, and one with coronary thrombosis. The remaining 35 variants operate through mechanisms yet to be determined [3].

Most genome wide association studies (GWAS) for CAD have been performed on populations of European descent, but increasing numbers of such investigations are now performed also on populations of Asian or African ancestry. In the literature currently available, some of the genetic effects observed are shared among ethnic groups but many times genetic effects are different across populations of different ancestry. The latter is the case of the 9p21 locus, which has been documented in Europeans and also in other ethnic groups including East and South Asians, but not among African Americans [4]. Demography and selection are the most important evolutionary forces that shape the population-specific patterns of mutations and linkage disequilibrium (LD) identified across populations of different ancestry. Demographic phenomena such as population expansions, subdivisions and bottleneck events could underlie differences among populations in a uniform way across genes. On the other hand, there are evolutionary processes with locus-specific effects. The genetic basis of common complex diseases may have partially been shaped by positive selection events, which simultaneously increased fitness and susceptibility to the disease. An additional difficulty to detect the role of selection is that methods to detect it have historically been challenged by the confounding effects of demography [5]. In any case, examples of positive selection have been identified in complex diseases such as type 1 diabetes, rheumatoid arthritis, or Crohn’s disease [6].

Concerning CAD risk regions, in the current literature there are discrepancies regarding the role of natural selection. A recent survey proposed natural selection as a possible explanation for the observed differences between Africa, East Asia, America, and Oceania in risk allele frequencies (RAFs) for eight CAD risk single nucleotide polymorphisms (SNPs) [7]. Another study reported evidence for selection at several SNPs identified through GWAS on sets of genes implicated in cardiovascular diseases [8]. In addition, Soranzo et al. identified one haplotype in a region of long-range LD (12q24) that contains disease loci for CAD, hypertension and type I diabetes, and that recently spread by positive selection in Europeans [9]. On the contrary, a recent survey affirmed that genetic differences for CAD among world-wide populations are due to random and demographic processes [6]. However, the conclusions of this last paper were not demonstrated through the use of different methodological approaches. Indeed, the authors used two tests (iHS and LRH) exclusively based on the same class of analytical method (LD decay) to search for signals of selection in CAD risk loci. Despite all the studies performed so far, the genetic architecture of CAD is not completely understood yet. In CAD, as in any complex trait, risk variants at many different loci may contribute to the phenotype each with a small effect. By combining evidence from GWAS with evidence from selection scans, it may be possible to separate true causative regions from the background noise inherent in GWAS [8]. The role that natural selection plays in CAD population differentiation may be a good tool to improve the knowledge of this complex trait.

Population differences in risk loci associated to CAD were recently detected for the 1p13, 1q41, 9p13, and 10q11 regions. These genomic regions showed disparities in the specific risk markers associated with CAD between European and North African samples [10].

The present work is centred on these four genetic regions (1p13, 1q41, 9p21 and 10q11) to explore whether the population genetic diversity is better explained by demographic or by natural selection effects.

To do this, we have analysed these risk regions in a set of North European and Mediterranean populations. Several specific tests to detect positive and/or balancing selection were performed. In addition, these analyses were expanded to populations from Asia and Africa looking for signatures of selection shared across continents or belonging to a specific population group.

## Materials and Methods

### Ethics statement

The study has been specifically approved by the Ethical Committee of the University of Barcelona (Institutional Review Board: IRB00003099) and all the participants provided a written informed consent.

### Sample description

Nineteen population samples from Europe, Middle East and North Africa were genetically tested. DNA samples of 868 healthy unrelated individuals of both sexes having their relatives for at least three generations born in the same geographical region were analyzed.

**Fig 1:**
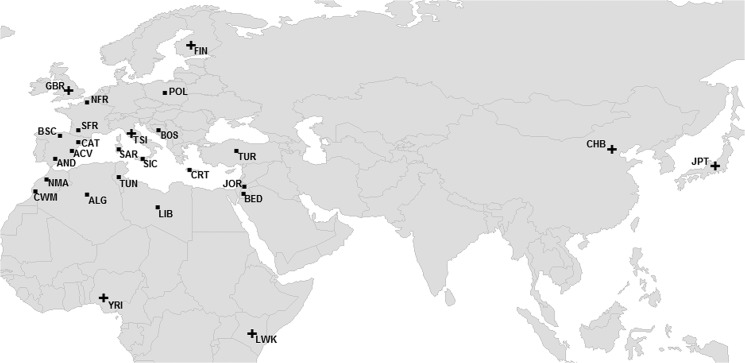
Geographic distribution of European, African and Asian samples. See S1 Table for abbreviation codes. Symbols: “▪” samples genotyped in this study, “+” data from the 1000 Genomes Project.

Population details of all samples, including sample size, geographic origin and coordinates are recorded in S1 Table. The geographic distribution of the analyzed samples is displayed in Fig 1. Europe is represented by samples from Poland, Spain (4 populations), France (2 populations), Italy (2 populations), Bosnia-Herzegovina, Greece, and Turkey. The Middle East is represented by two populations from Jordan and the North African area is represented by samples from Tunisia, Morocco (2 populations), Algeria and Libya. Genetic data from European (CEU, GBR, FIN, and TSI), Sub-Saharan African (LWK and YRI) and Asian (JPT and CHB) samples from the 1000 Genomes Project [11] were also included in the analyses (S1 Table).

### Polymorphisms and genotyping

Genomic DNA was extracted from blood cells using a Blood Midi kit (Omega Biotek, USA) according to manufacturer's procedures. DNA samples were genotyped for a combined set of 384 SNPs using a Custom GoldenGate Panel (Illumina Inc., San Diego, CA). These polymorphisms were located in four loci previously associated and replicated in independent studies with CAD [12, 3, 13, 14, 15], specifically the 1p13 (61SNPs), 1q41 (38 SNPs), 9p21 (159 SNPs), and 10q11 (126 SNPs) chromosomal regions.

SNPs were selected as a representative set of the common variation in the four genomic regions, according to the following criteria: i) average coverage of 1 SNP every 1.5 kb, ii) minor allele frequency (MAF) higher than 0.05 in CEU and TSI HapMap populations, iii) given priority to markers not in linkage disequilibrium (LD) (r^2^<0.8) in European populations, and iv) prioritizing markers previously associated with CAD [4]. These criteria were applied giving preference to tag SNPs. Genomic location of genetic variants is shown in S2 Table.

In addition, all the variation concerning these four genomic regions reported in the 1000 Genomes Project was used to perform three specific selection analyses: Tajima’s D, Long Range Haplotype (LRH) test and F_ST_ tests.

### Data Cleaning and Quality Control

Genotyping rate per SNP and individual, cryptic relatedness, and LD pruning (r^2^≥0.8) were assessed using PLINK version 1.07 [16]. Individuals with more than 5% of missing genotypes were eliminated. Non-polymorphic SNPs or SNPs with genotyping rate lower than 0.95 were also removed from the analyses.

Population allele frequencies and heterozygosity calculations were performed using *PopGenkit* R software package [17]. Fitting to Hardy-Weinberg equilibrium in each population was calculated by means of Arlequin v3.5 software [18].

### Population Structure Analyses

Genetic structure was assessed by molecular variance (AMOVA) using Wright's F-statistics in the European and Mediterranean area and, in a broader context, in the European, African and Asian continents. Populations were clustered according to geographic criteria. Middle Eastern samples were clustered into the South European group due to affinities in their genetic distances. The samples were clustered in: 1) North Europe, South Europe, and North Africa, and 2) North Europe, South Europe, North Africa, Sub-Saharan Africa and Asia. AMOVAs were carried out using Arlequin v3.5 [18].

Genetic relationships among populations were assessed by Reynolds genetic distance and by a principal component (PC) analysis calculated through the *Adegenet* [19] and *FactoMineR* R packages [20].

The LD blocks based on the LD measure D′ confidence interval [21] were performed in the four genomic regions using Haplot software [22]. These analyses were performed twice: first, considering all markers in all samples and, second, taking into account only the chromosomal fragments where markers previously associated in Europeans [21, 13, 22], Africans [10, 23, 24] and Asians [25] are located. Permutation procedures to obtain a Monte Carlo statistical significance were used for each chromosomal region in order to evaluate population differences in LD structure through the VarLD software version 1.0 [26]. This analysis was carried out using 150 randomly selected individuals for each different population group (North Europe, South Europe, North Africa, Sub-Saharan Africa, and Asia) to avoid biases due to differences in sample size. The LD statistics (D’ and r^2^) for each pair of risk SNPs in each population analyzed were calculated through the Haploview software version 4.2 [27].

### Selection Analyses

Detection of potential loci under selection was assessed using different methods. The first method used is based on the probability of observing locus by locus AMOVA statistics as a function of heterozygosity, given a null distribution generated under a hierarchically-structured island model of population differentiation [28]. This test detects loci under selection from genome scans that contrast patterns of genetic diversity within and between populations. A total of 20000 coalescent simulations in 50 groups, with 100 simulated demes per group were performed. The observed locus-specific measures of population differentiation (F_ST_) were compared to a null distribution obtained by simulation samples. The P-value of each locus was estimated from the joint distribution of heterozygosity and F_ST_ using a kernel density estimation procedure. The null distribution generated was summarized by quantiles of the joint distribution. The 1% and 99% quantiles of the distribution correspond to markers potentially under balancing or directional selection, respectively, at the 1% level, without multiple-test correction and assuming one-tailed test. This method was performed using Arlequin v3.5 software [18].

Moreover, the spatial ancestry (SPA) analysis was used to detect positive signatures of selection [29]. In this analysis, SNP data were used to model allele frequency distributions in a geographic space. Applying the SPA approach, polymorphisms showing steep geographic gradients in allele frequencies can be identified through SPA scores reflecting the steepness of the geographic gradient. Large SPA scores are indicators of potential selection. In this study we focused on SPA scores above the 99th percentile, based on the 367 SNPs analysed. In order to detect continental adaptation events, analyses were performed only in Europe and North Africa. Asian and Sub-Saharan African samples were not tested for the SPA scores due to the low number of available populations for these geographic regions.

Spatial distribution of mean MAF for one of the three CAD SNPs potentially positively selected across populations was mapped using the geostatistical method known as kriging from the ArcGIS software (ESRI, Redlands, CA, USA). Since anisotropy was not detected in the semivariogram, we used the ordinary spherical interpolation kriging method [30]. Finally, the Tajima’s D [31], the extended haplotype homozygosity (EHH) and the cross-continental F_ST_ tests were conducted in three populations of the larger dataset of the 1000 Genomes Project (CEU, CHB, and YRI) through the web tool 1000 Genome Selection Browser 1.0 [32]. The Tajima’s D test was performed using windows of 3-kb to search signatures of balancing selection. The EHH test was applied to validate loci under positive selection found in previous tests, using a larger chromosome region of 20-Mb. The significance of the three tests was assessed by the rank score tracks, which provided a comparison to the rest of the genome, calculated by means of the web tool 1000 Genome Selection Browser 1.0 [32]. They were calculated by empirical comparisons sorting all the scores genome-wide and determining the-log10 of the rank divided by the number of values in the distribution, taking the upper tail for the F_ST_ and the EHH tests, and the lower tail for the Tajima’s D test. The p-values were assigned on the basis of the rank score tracks. In this study, Tajima’s D, EHH average and F_ST_ scores above the 95th percentile of the top extreme genome-wide distribution were considered as significant. All p-values were corrected for multiple comparisons applying the false discovery rate (FDR) method [33] by means of the *stats* R package [17].

## Results

### Genotyping and quality control

After pruning, the average coverage was 1 SNP every 1.8 Kb. Genotyping rate for the 384 SNPs initially tested was 95.8%. Sixteen SNPs were not successfully genotyped and were removed from the study. One SNP showed a significant departure from Hardy-Weinberg equilibrium after Bonferroni correction. Thus, a total of 367 markers were included in the analyses after quality control: 59 SNPs in 1p13 (S1 Fig), 37 in 1q41 (S2 Fig), 155 in 9p21 (S3 Fig), and 116 in 10q11 (S4 Fig). Moreover, twenty individuals were removed for low genotyping rate (>5% of missing genotypes); consequently, 848 population samples were analysed. Individual genotypes are included in S1 Dataset. For PC and for Reynolds genetic distance analyses, a total of 176 SNPs were removed due to high LD (r^2^>0.8) hence, only 190 SNPs were included in population relationship analyses.

### Levels of diversity and population relationships

The SNP minor allele frequencies (MAF) in population samples and global heterozygosities per marker and population are presented in S2 Table. Mean heterozygosities per SNP were moderate-high, ranging from 0.038 to 0.495. Per population, heterozygosities ranged from 0.259 to 0.345.

Hierarchical AMOVA estimates when populations were clustered in North Europe, South Europe, North Africa, Sub-Saharan Africa and Asia were F_ST_ = 0.085 and F_CT_ = 0.080 (both p<0.0001). When Sub-Sahara African and Asian data were removed and the remaining populations were grouped in North Europe, South Europe and North Africa, AMOVA estimates were F_ST_ = 0.017 and F_CT_ = 0.012 (both p<0.0001). These values indicate a statistically significant geographic structure of genetic variation in the three continents, and also across Europe and North Africa.

Population genetic distances are indicated in S3 Table. The highest average genetic distance was among North African and European samples (0.170±0.031). The genetic distance between Middle East and North Africa was higher (0.156±0.032) with respect to Europe (0.141±0.023).

**Fig 2:**
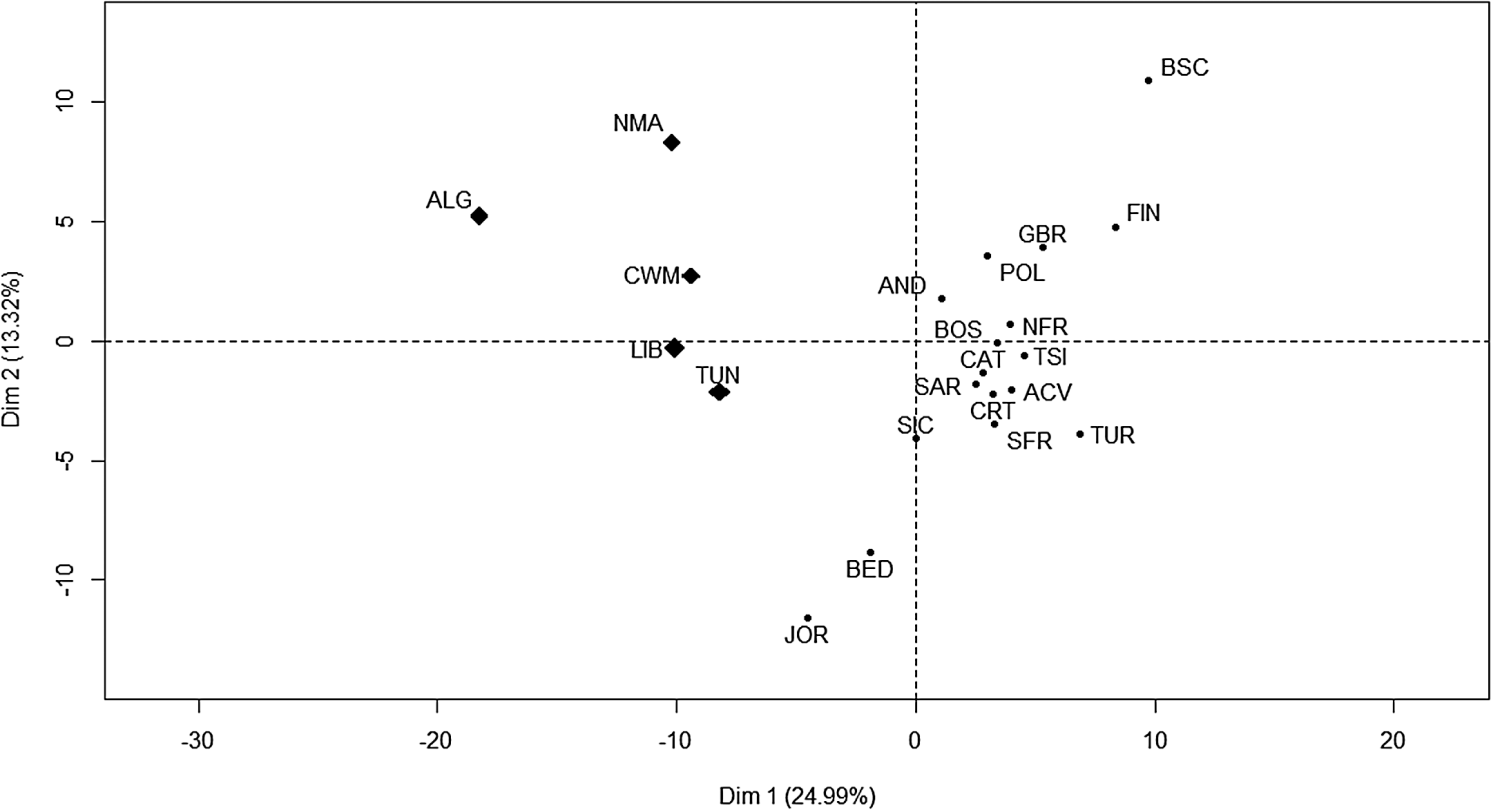
PC plot of the 22 population from Europe (circle), Middle East (circle) and North Africa (diamond) based on the variation of 190 independent SNPs located in the CAD risk regions 1p13, 1q41, 9p21 and 10q11.

Concerning population relationships, in the PC analysis (Fig 2) of the European, North African and Middle East populations, the two first axes accounted for 38.31% of the total genetic variance. In the first axis (24.99% of the total variance) North African samples appeared clearly separated from the European ones. The second component underlined the separation of the Basque Country on one side, and the Middle Eastern samples on the other.

### LD analyses

Different LD blocks were identified among populations. At first glance, Asian and Sub-Saharan African samples showed different and characteristic LD patterns when compared with other continental populations (S5–S8 Figs). Significant differences in LD structure for the 4 genomic regions were observed in pairwise comparisons between Europe, Africa, and Asia (Table 1). A more detailed analysis within continents indicated that only the 9p21 region showed significantly different LD patterns in all subcontinental groups. Between North and South Europe the LD blocks were not significantly different in the regions 1p13, 1q41 and 10q11.

**Table 1:** LD difference p-values for the 4 genomic regions analysed. Pairwise comparisons between North Europe (NEUR), South Europe (SEUR), North Africa (NAFR), Sub-Saharan Africa (SAFR), and Asia.

**1p13**	**NEUR**	**SEUR**	**NAFR**	**SAFR**	**1q41**	**NEUR**	**SEUR**	**NAFR**	**SAFR**
**SEUR**	0.1740	-			**SEUR**	0.6001	-		
**NAFR**	0.0224	0.0005	-		**NAFR**	0.0016	0.0156	-	
**SAFR**	0.0096	0.0006	0.0328	-	**SAFR**	0.0001	0.0001	0.0001	-
**ASIA**	0.0001	0.0001	0.0001	0.0001	**ASIA**	0.0489	0.1060	0.0034	0.0001
**9p21**	**NEUR**	**SEUR**	**NAFR**	**SAFR**	**10q11**	**NEUR**	**SEUR**	**NAFR**	**SAFR**
**SEUR**	0.0179	-			**SEUR**	0.1193	-		
**NAFR**	0.0001	0.0001	-		**NAFR**	0.0001	0.0128	-	
**SAFR**	0.0001	0.0001	0.0001	-	**SAFR**	0.0001	0.0001	0.0001	-
**ASIA**	0.0001	0.0001	0.0001	0.0001	**ASIA**	0.0144	0.0032	0.0007	0.0001

Focusing only on SNPs previously associated with CAD, LD statistics (D’ and r^2^) for each pair of risk SNPs and for each analysed population are provided in S4 Table. Risk SNPs in the same haplotype block (red colour in S4 Table) showed a high degree of variation in LD (0.35≤ D’≤1) in the populations analysed. The haplotype block information for these markers is provided in S5–S7 Tables for the 1p13, 9p21 and 10q11 regions, respectively.

**Fig 3:**
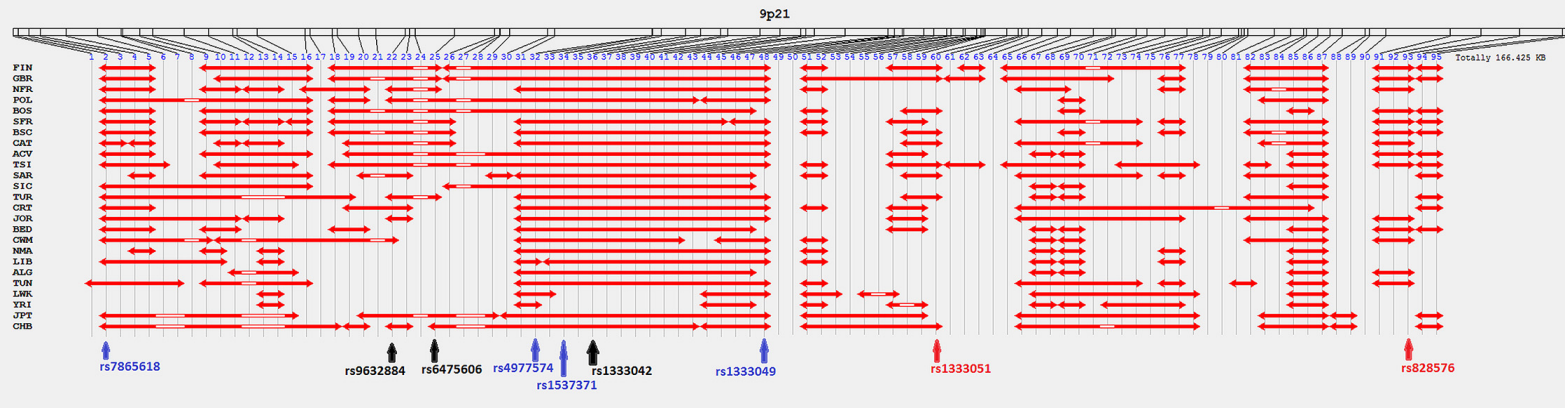
LD blocks for the region 9p21 in 26 populations from Europe, Africa and Asia. Markers previously associated with CAD only in Europe are highlighted in blue, in Caucasians together with African Americans and Chinese in black and in North Africa in red.

**Fig 4:**
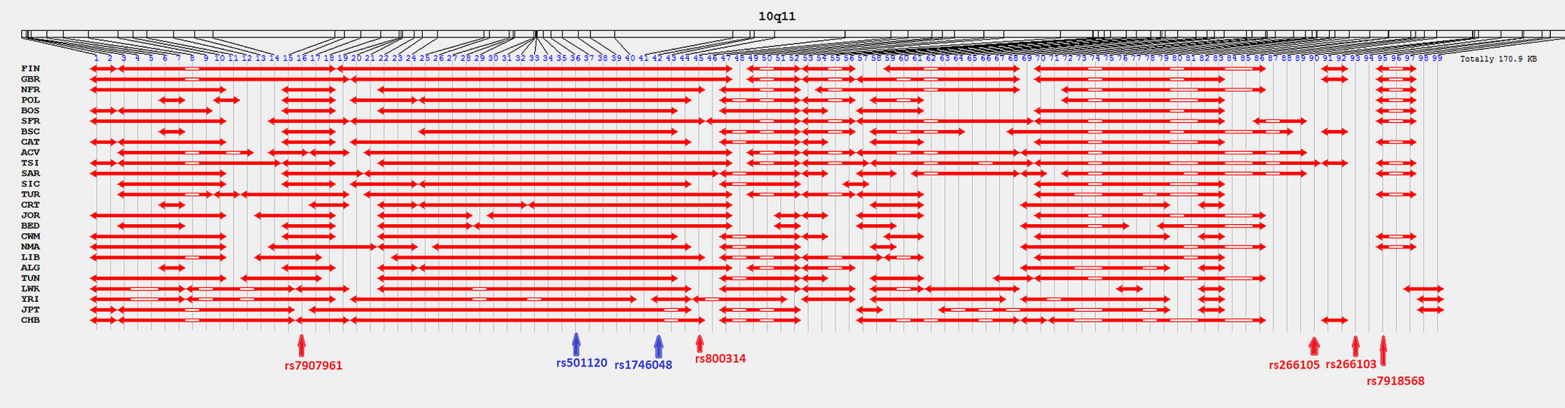
LD blocks for the region 10q11 in 26 populations from Europe, Africa and Asia. Markers previously associated with CAD only in Europe are highlighted in blue and in North Africa in red.

The most remarkable differences in LD block structure were present in the 9p21 (Fig 3) and 10q11 regions (Fig 4). It is interesting to note the different positions shown by CAD associated markers in Europe and North Africa. In the 9p21 region, associated markers in Europe (blue) and in Caucasians together with African Americans or Chinese (black) laid at the beginning of the region, whereas markers associated in North Africa (red) were located at the end (Fig 3). In this region, the risk SNPs rs9632884, rs6475606, rs4977574, rs1537371, rs1333042, and rs1333049, located in the same haplotype block, were in tight LD in the European, North African and Middle Eastern populations studied (0.831≤ D’≤1) (S4 Table). On the other hand, in the Sub-Saharan African, and Asian samples LD values ranged from 0.091≤ D’≤1 (grey in S4 Table). These SNPs were located in different haplotype blocks except in Tuscany and Andalusia (S6 Table).

In the 10q11 genomic region, the three risk SNPs analysed (Fig 4) presented LD values in the range of 0.772≤ D’≤1 in all the populations studied except in Sub-Saharan African and Asian samples (0.035≤ D’≤1) (S4 Table). These three risk markers were located in the same haplotype block in all the populations analysed except in Yoruba samples (S7 Table). Regarding the 1p13 region (S9 Fig), the two risk markers, rs599839 and rs646776, presented high LD values in all the populations studied (D’>0.888) (S4 Table). These two risk factors were located in the same haplotype block except in African populations (S5 Table).

The 1q41 genomic region showed previous associations with CAD only in samples of European origin and not in North Africans [10]. The most representative associated SNP in this region was the rs17465637 [3] (S10 Fig).

### Detection of potential signals of selection

The results of the locus by locus F_ST_ statistics and their significance as potential indicators of selection are presented in S8 Table. In the European/Mediterranean area, 16 loci were potentially under selection (i.e. loci with significantly low or high F_ST_ values given their heterozygosity). Out of these 16 loci, seven had high F_ST_ values (F_ST_ range = 0.067–0.094) indicating potential positive selection and the other nine showed low F_ST_ values (F_ST_ range = <0.0001–0.0004) suggesting balancing selection. Regarding cross-continental analyses, 15 loci were potentially under selection, eight of which indicated potential positive selection (F_ST_ range = 0.248–0.293) and the other seven suggested balancing selection (F_ST_ range = <0.0001–0.007).

**Fig 5:**
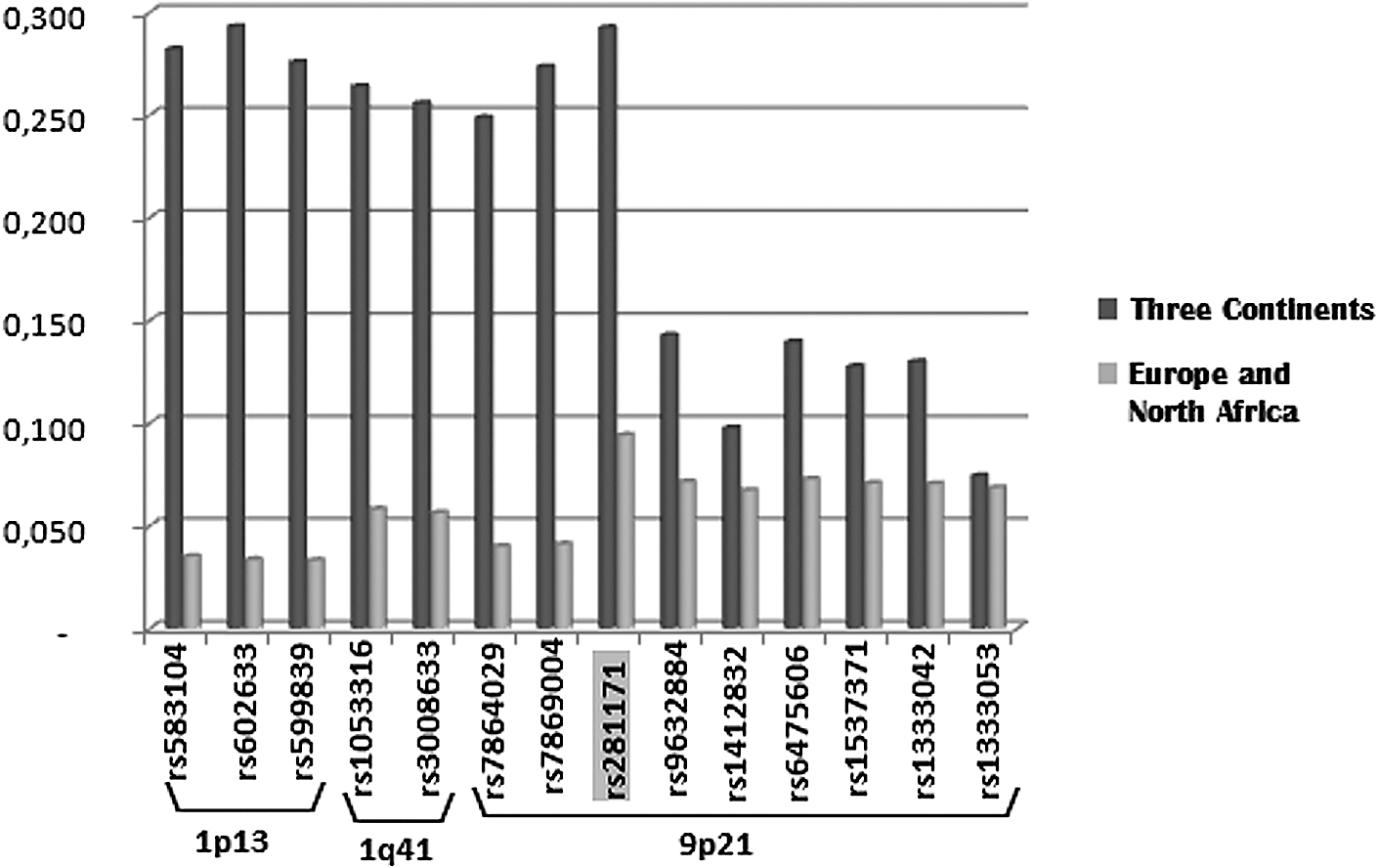
Plot of F_ST_ values of the markers under potential positive selection found in the F_ST_ vs heterozygosity analysis. On the left markers found only in the context of the three continents, on the right markers found only in Europe and North Africa and in the middle (grey) the SNPs identified in both analyses.

The potential positively selected markers found in the above analyses (F_ST_ vs heterozygosity) were a total of 14 and were located in regions 1p13, 1q41, and 9p21. Out of these 14 SNPs, seven were found only in the context of the three continents, six only in Europe and North Africa, and one in both groups (rs2811717 in the 9p21 region) (Fig 5). Fifteen markers located in chromosomes 9 and 10 were potentially under balancing selection: six in the context of the three continents, eight in Europe and North Africa and one in common (rs17155733 in the 10q11 region). The average allele frequency of these markers in the analysed populations is of 0.50±0.35 (S2 Table). Consequently the possible influence of negative selection can be discarded.

**Fig 6:**
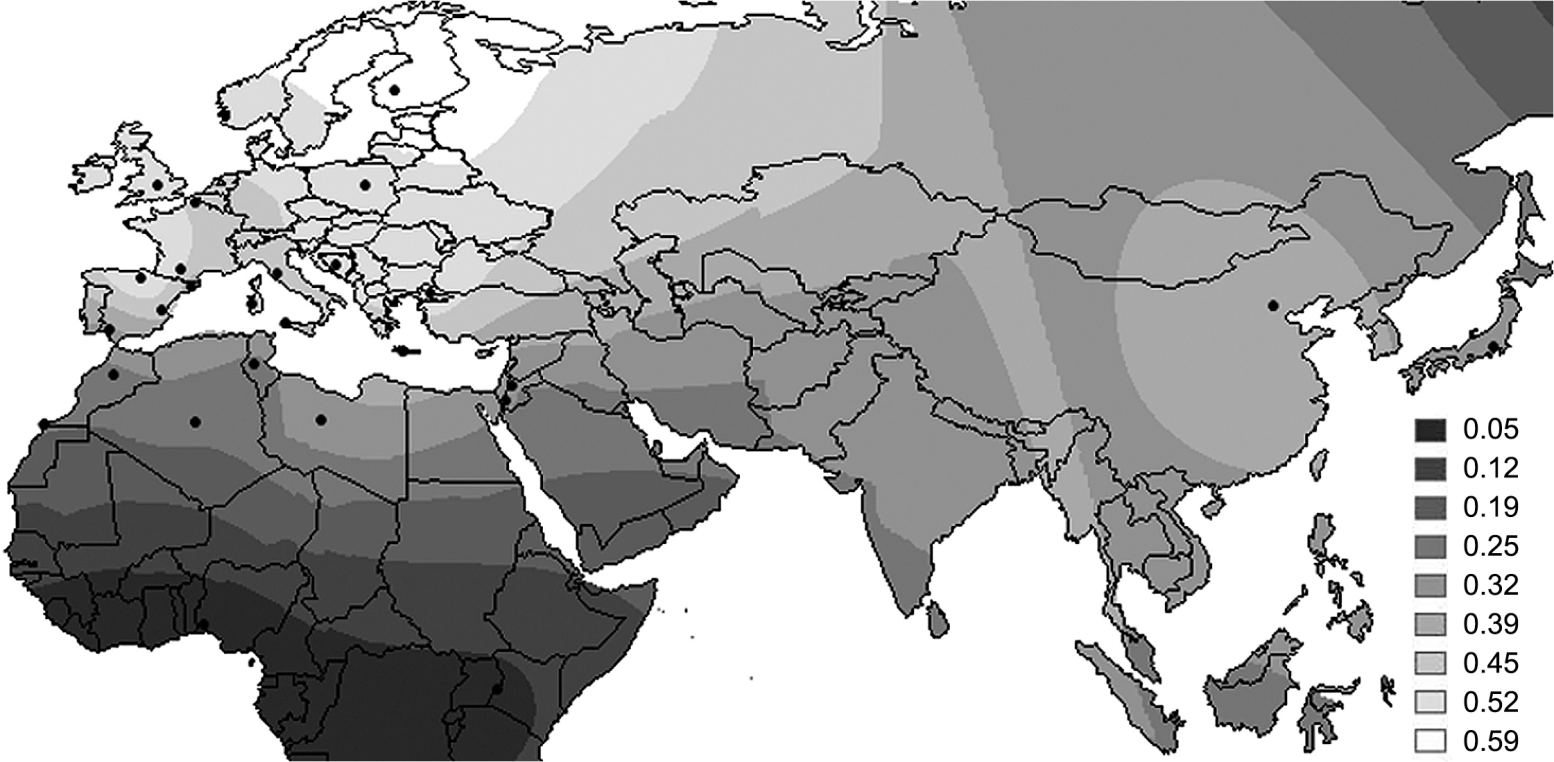
Contour map of the MAF of the CAD risk SNP rs1333042 potentially positive selected in the 9p21 region.

In the SPA analysis, three SNPs (rs9632884, rs1537371, and rs1333042) showed SPA scores above the 99th percentile in each one of the sample group: North Africa (SPA value in the 99th percentile: 0.87) and Europe (1.95) (S8 Table). None of these three North African markers showed association with CAD in previous studies or was identified as being under potential selective pressure in the previous tests. On the contrary, all three markers with the highest SPA score in Europe were among the SNPs that showed signs of positive selection in Europe and North Africa in the F_ST_/H_O_ tests. Also, these SNPs have been associated with CAD in previous studies [34, 35, 36] and are located in the 9p21 genomic region: the CAD risk locus most studied [37]. These three risk markers are in strong LD in most populations analysed (S4 Table). A geographical distribution of the MAF of one of these three SNPs, the rs1333042, in the populations studied is represented in a smoothed spherical contour map in Fig 6.

The cross-continental F_ST_ and the EHH average tests, performed with the CEU, CHB and YRI samples from the 1000 Genomes Selection Browser, pointed out markers in the extreme 5% of the genome-wide distribution. Markers with high F_ST_ values indicating potential positive selection (top 2.5%) were identified in the four regions analysed (62 markers in the 1p13, 32 in the 1q41, 121 in the 9p21, and 63 in the 10q11). Markers with extremely low F_ST_ values (bottom 2.5%), indicating potential signatures of balancing selection, were also detected in all the regions studied (13 in the 1p13, 3 in the 1q41, 42 in the 9p21, and 53 in the 10q11) (S9 Table). After applying correction for multiple testing, seven markers with extremely high F_ST_ values in the 9p21 region maintained their significance (red in S9 Table). These markers were located in an 11-kb region (chr9: 21932366–21933125) and are not included in the 367 SNPs analysed in the 26 populations. Comparing the F_ST_ estimated in the 26 populations analysed with the 1000 Genomes Browser Data, it is noteworthy the common signals detected for the rs2811717 in the 9p21 region (S8 Table). For this SNP the differences in cross-continental F_ST_ values detected in the 26 population studied compared to the 1000 Genomes Browser data (0.29 vs. 0.65, respectively) are likely due to the different sets of populations included in each group (S8 Table). Regarding the region 1p13, F_ST_ values of the three common markers detected in both analyses (rs602633, rs583104, and rs599839) are very different comparing the 1000 Genomes data for CEU, CHB and YRI (0.57–0.59) with the 26 populations analysed (0.27–0.29). Also in this case, this is likely due to the different populations included in the analysis, mainly in the African continent (YRI alone versus 5 North African and 2 Sub-Saharan African populations together). The EHH average test, used to validate the results found in the 9p21 region, showed potential signals of positive selection (p≤0.05) in the three populations analysed (grey in S10 Table). It is noteworthy the fact that the CAD risk marker rs1333042, which showed potential signals of positive selection in the previous tests performed (F_ST_ vs H_O._ and SPA score), is located in one of the 3-kb regions that show significant EHH values (region highlighted in grey in S10 Table). However, no EHH average showed significant signals of positive selection after correcting for multiple comparisons. Finally, concerning potential signatures of balancing selection, the Tajima’s D analysis pointed out ten, five, and fourteen windows with Tajima's D values above the 95th percentile (p≤0.05) of genome-wide distributions in CEU (9p21), CHB (10q11) and YRI (10q11), respectively (S8 Table). Two regions were found to overlap, a first between Asia and Sub-Saharan Africa in 10q11, for a 15 kb extension and a second one across European samples in 9p21 for a 24 kb (S8 Table). Even though, after applying corrections for multiple comparisons no windows showed significant signals of balancing selection.

The most relevant findings of positive selection regarding the 1p13, 1q41, and 9p21 genomic regions, and of balancing selection of the regions 9p21 and 10q11 are summarized in Tables 2 and 3.

**Table 2:** Significant positive selection results in a minimum of two different tests. Observed (Obs) F_ST_ with selection significance (highlighted P value<0.01) for the three continents (Africa, Asia and Europe) and for Europe and North Africa. Global cross-continental F_ST_ scores in the CEU, CHB, and YRI samples from the 1000 Genomes Selection Browser. Significant high and low FST values (extreme top 5% values of the distribution of F_ST_ values across the genome) are highlighted in bold. P values were calculated on the basis of higher rank scores in the genomic distribution. SPA scores for continental European, and North African allele gradients (highlighted in bold SPA scores above 99th percentile).

			Three Continents		Europe and North Africa		1000 Genomes Browser		Europe	North Africa
Genomic region	Position	SNP_ID	Obs FST	FST P-value	Obs FST	FST P-value	FST	P_values	SPA scores	SPA scores
**1p13**	**109821307**	**rs583104**	0.282	**0.004**	0.035	0.136	0.594	**0.001**	0.655	0.300
**1p13**	**109821511**	**rs602633**	0.293	**0.002**	0.034	0.151	0.592	**0.001**	0.596	0.312
**1p13**	**109822166**	**rs599839**	0.276	**0.005**	0.033	0.160	0.578	**0.001**	0.651	0.278
**1q41**	**222839838**	**rs1053316**	0.264	**0.005**	0.058	0.019	0.416	**0.008**	0.376	0.534
**1q41**	**222844840**	**rs3008633**	0.256	**0.007**	0.056	0.023	0.412	**0.009**	0.310	0.484
**9p21**	**21920346**	**rs4977746**	0.236	0.011	0.051	0.032	0.342	**0.018**	0.425	**1.116**
**9p21**	**21930147**	**rs7864029**	0.249	**0.007**	0.040	0.085	0.350	**0.017**	0.373	0.517
**9p21**	**21931896**	**rs7869004**	0.273	**0.004**	0.041	0.077	0.389	**0.011**	0.480	0.592
**9p21**	**21946322**	**rs2811717**	0.292	**0.003**	0.094	**0.001**	0.648	**4.923E-04**	0.310	0.624
**9p21**	**22072301**	**rs9632884**	0.143	0.117	0.071	**0.008**	0.327	**0.021**	**2.075**	0.358
**9p21**	**22099568**	**rs1537371**	0.128	0.159	0.071	**0.008**	0.292	0.029	**2.046**	0.435
**9p21**	**22103813**	**rs1333042**	0.130	0.152	0.070	**0.008**	0.287	0.031	**2.043**	0.464

**Table 3:** Significant balancing selection results in a minimum of two different tests. Observed (Obs) F_ST_ with selection significance (highlighted P value<0.01) for the three continents (Africa, Asia and Europe), and for Europe and North Africa. Global cross-continental F_ST_ scores and Tajima’s D test results in the CEU, CHB, and YRI samples from the 1000 Genomes Selection Browser. Significant high and low F_ST_ values (extreme top 5% values of the distribution of F_ST_ values across the genome), and signals of potential balancing selection (P value≤0.05) are highlighted in bold. Both F_ST_ and Tajima D P values were calculated on the basis of higher rank scores in the genomic distribution.

				Three Continents		Europe and North Africa		1000 Genomes Browser		CEU		CHB		YRI	
Genomic region	chromStart	chromEnd	Significant SNPs in the window	Obs FST	FST P-value	Obs FST	FST P-value	FST	P_values	TajimaD	P_values	TajimaD	P_values	TajimaD	P_values
**9p21**	**22110605**	**22113605**								1.812	**0.044**	1.534	0.105	-0.878	0.776
**9p21**	**22113605**	**22116605**	**rs2383206**	0.028	0.075	0.018	0.394	-0.004	**0.997**	1.984	**0.030**	1.335	0.148	-0.756	0.705
**9p21**	**22116605**	**22119605**								1.819	**0.044**	1.198	0.183	-0.830	0.750
**9p21**	**22119605**	**22122605**								1.935	**0.034**	1.309	0.154	-0.721	0.684
**9p21**	**22122605**	**22125605**								1.947	**0.033**	1.227	0.175	-0.593	0.600
**9p21**	**22125605**	**22128605**								2.019	**0.028**	1.240	0.171	-0.451	0.503
**9p21**	**22128605**	**22131605**								2.056	**0.026**	1.056	0.223	-0.305	0.406
**9p21**	**22131605**	**22134605**	**rs1751449**	0.017	0.030	0.000	**0.005**	-0.005	**0.998**	1.837	**0.042**	0.980	0.247	-0.437	0.494
**10q11**			**rs11597731**	-0.001	**0.005**	-0.002	0.121	-0.003	**0.995**						
**10q11**	**44715700**	**44718700**	**rs2209067**	0.001	**0.001**	0.002	0.036	-0.001	**0.984**	0.194	0.507	0.932	0.262	-0.402	0.471
**10q11**			**rs17155733**	-0.000	**0.005**	0.000	**0.003**	0.002	0.748						
**10q11**	**44739700**	**44742700**								0.164	0.520	1.708	0.078	0.692	**0.046**
**10q11**	**44742700**	**44745700**								0.053	0.565	1.762	0.070	0.913	**0.026**
**10q11**	**44745700**	**44748700**								-0.050	0.606	1.757	0.071	0.959	**0.023**
**10q11**	**44748700**	**44751700**								-0.108	0.629	1.999	**0.044**	0.846	**0.031**
**10q11**	**44751700**	**44754700**								-0.087	0.621	2.102	**0.036**	1.008	**0.020**
**10q11**	**44754700**	**44757700**								0.011	0.582	2.209	**0.029**	1.081	**0.017**
**10q11**	**44757700**	**44760700**								-0.017	0.593	2.027	**0.042**	1.165	**0.013**
**10q11**	**44760700**	**44763700**								-0.153	0.645	1.964	**0.048**	1.185	**0.013**
**10q11**	**44763700**	**44766700**								-0.312	0.702	1.862	0.058	1.073	**0.017**
**10q11**	**44766700**	**44769700**								-0.545	0.774	1.490	0.115	0.844	**0.031**
**10q11**	**44769700**	**44772700**	**rs266088**	0.007	**0.009**	0.002	0.037	-0.002	0.990	-0.619	0.794	1.187	0.186	0.688	**0.047**

## Discussion

This study explores for the first time the genetic diversity in four genomic regions associated with CAD (1p13, 1q41, 9p21, and 10q11) through the genotyping of a set of European and Mediterranean populations and using European, Asian and Sub-Saharan African data from the 1000 Genomes Project [11]. The main aim was to evaluate the relative importance of demography and natural selection as a cause of population variability in some CAD risk regions identified in previous GWAS.

Diversity population indices indicate significant genetic structure in the context of the three continents (F_ST_ = 0.085; p<0.0001) and also in Europe and in the Mediterranean area (F_ST_ = 0.017; p<0.0001). The degree of genetic diversity observed in these four regions between continents is comparable to that obtained from genome-wide genotyping and sequencing studies [11, 38]. Moreover, in the European and Mediterranean context, these regions of epidemiological importance exhibit the same genetic variation pattern described using sets of neutral markers (e.g. Alu insertion polymorphisms) and genome-wide arrays in previous population studies [39, 40, 41, 42].

Regarding the LD haplotype blocks, this survey pointed to high levels of variation in LD and sometimes to a different chromosomal location of CAD risk markers in populations of different ancestry. The significant differences in LD structure found across European, North African, Asian, and South African samples likely explain population differences in markers associated to CAD across populations. These LD differences can be explained by the previously observed trend of lower haplotype diversity and higher LD values when the geographic distance from Africa increases, [43], in agreement with the Recent-African-Origin hypothesis [44].

Even though the general patterns of genetic variation are in agreement with the genetic structure generated by demographic processes, we also observed potential signals of positive and balancing selection in some specific markers and haplotypes. Seven SNPs under potential positive selection showed association to diseases in previous studies [10, 24, 34, 35, 36, 45, 46, 47], whereas none of the markers under balancing selection was identified as a risk factor in previous GWAS. Regarding the seven risk markers under positive selection, three of them are located in the 1p13 region and four in 9p21. In the 1p13 region, two of the three markers (rs602633 and rs583104) were associated with LDL-C [45, 46]. The other positively selected SNP, rs599839, was associated with CAD in Caucasian populations [24]. These three markers were detected in two of the selection tests performed (F_ST_ vs H_O_ with 367 markers and F_ST_ with the 1000 Genomes Selection Browser) in the context of three continents. The F_ST_ value detected in the 1000 Genomes Data is in the same order of magnitude than recent positive selection signals (F_ST_>0.5) detected in previous studies performed on the lactase gene [48] and on genome-wide scans [49].

The 9p21 genomic region, the most complex and studied CAD risk region [3], showed consistent signals of positive selection. Indeed positive signatures of selection were identified in each one of the four tests performed (F_ST_ vs H_O_, SPA score, F_ST_ and LRH test). The consistency for selection signals in the 9p21 genomic region lies in the fact that these different tests are all based on the same aspect of the data, i.e. population differentiation. Briefly, four variants showed evidence of positive selection: rs9632884, rs6475606, rs1537371 and rs1333042. These SNPs were previously associated with CAD [34, 35, 36, 47] and showed slightly different patterns of LD blocks across populations.

Thanks to the high density of populations available for Europe and North Africa, we were able to do a spatial analysis of selection (SPA test) in these geographic areas. Thus, the positive signals of selection observed in the F_ST_ vs H_O_ tests could be mainly attributed to Europeans. In fact, the European SPA scores are higher than those detected in North Africans. Additionally, the confirmation of three markers in the 9p21 genomic region (rs9632884, rs1537371, and rs1333042) in two tests (F_ST_ vs H_O_ and SPA score in Europe) is a good indicator of consistency across our results. As an independent replication using the 1000 Genomes dataset, the region containing one of these 3 markers (rs1333042) has shown a significant EHH value (before correction for multiple testing) in the LRH test. Moreover, this SNP, used as representative of the other two risk markers, shows a South to North gradient of increase in MAF. The gradient observed is in agreement with CAD incidence [50] and with previous genetic studies based on the apolipoprotein E4 [51], or the genetic risk score of nitric oxide synthases [52], both associated with susceptibility to CAD. The South to North frequency gradient, in accordance with CAD incidence rates, may be correlated with a potential selective role of CAD in the configuration of genetic diversity in current human populations. On the contrary, another study showed a geographical pattern opposite and uncorrelated with the disease incidence for genetics variants correlated with CAD [53]. The observed North to South cline in frequency detected in that work was likely due to the spatial distribution of the whole genome variation present in the European continent, mainly shaped by demography. The potential positive selection signals of the 9p21 region were also detected using the 1000 Genomes data. In fact, seven markers of this region passed the multiple testing corrections in the F_ST_ tests showing F_ST_ values >0.78. In addition is noteworthy the potential positive signal detected for the rs2811717 in the 26 population studied and also in the 1000 Genomes Browser data. The F_ST_ value of this SNP in Europe (0.094) is substantially higher than 0.028, value used to identify the most prominent genome-wide candidate regions in a recent selection scan in Europe [54].

Regarding signatures of balancing selection, only the 9p21 and 10q11 regions showed evidence of balancing selections in the Tajima’s D and in the F_ST_ vs H_O_ tests. Although these regions did not pass the corrections for multiple testing, it is noteworthy that regions of 15 kb (encompassing five contiguous windows in 10q11) in Asian and African samples and 24 kb (corresponding to eight contiguous windows in 9p21) in European populations showed evidence of selection. The overlap in the signals observed for Asians and Africans in the 10q11 region suggests that this locus is likely subjected to selective processes in both populations, in agreement with Wang et al. [55], who estimated that 78% of selective events are shared by two or more populations. Regarding the 9p21 CAD risk region, the regions showing positive and balancing selection are physically separated by a minimum of 11kb (the last positive selected marker in the region: rs1333042 versus the first markers under balancing selection: rs2383206). The two different natural selection signals seem to have population specificity arguing for different evolutionary processes behind the positive and balancing selection in the same genomic region. While the plausible balancing selection signals found are acting mainly on European populations (Tajima’s D test), the signals of positive selections in the region 9p21 involve the three continents (F_ST_ and EHH tests).

The heterogeneity observed in the potential signals of natural selection in the different populations analyzed might be correlated with environmental variables. A recent whole genome scan reported evidence for selection on two markers implicated in cardiovascular disease by identifying the SNPs with the strongest correlation between allele frequencies and climate, specifically with winter solar radiation and summer precipitation rate in African and Western Eurasian populations [56]. Another study enumerated several genes involved in the causal pathways of atherosclerosis, that may be subject to various degrees of selective pressures resulting from climatic and dietary changes and host response to pathogens [57]. These pathways may influence the genetic susceptibility to CAD and the heterogeneity observed in the signals of selection among the populations analyzed.

Regarding CAD risk loci, the selection of harmful mutations may be due to the fact that they are in LD with a relatively strong still unknown beneficial polymorphism [6] that could be related to CAD. On the other hand, the positively selected loci could be related to other risk traits since many GWAS SNPs are associated with more than one trait [58]. In the case of the 9p21 locus, several genetic variants had been associated with multiple cancers [59], glaucoma [60], intracranial and abdominal aortic aneurysms [61], vascular dementia and late onset Alzheimer’s disease [62]. The response of a variant to selection is dependent also on the genetic background. Due to pleiotropy, the existence of selection at a specific locus may be related to different risk traits located in the same genomic region [58].

Although many selection scans have highlighted potentially interesting signals using genome-wide SNP data, it is currently difficult to assess how much confidence should be placed into them in the absence of clear signals of widespread, strong selection or of biological/functional information [63]. Recent data suggests that it is unusual for selection factors to drive new mutations rapidly to fixation in a specific population (the “hard sweep” model). A number of possible theories have been proposed to explain these potential genome-wide selective signals. Among others, most selection on individual alleles may be relatively weak so that alleles have not had time to sweep to fixation within continental populations. Moreover, the strength of selection may vary temporally, and it may be rare for selection to be consistently strong for the 10,000 years or more required to drive an allele close to fixation. Finally, much of human adaptation may proceed by either polygenic adaptation or soft sweeps that can be difficult to detect using standard methods [64]. This latter explanation (polygenic adaptation and/or soft sweeps) may be especially relevant for complex traits, such as cardiovascular diseases, in which several loci contribute to generate the disease. Current GWAS identified several common variants associated to CAD but at the moment there is an open debate about causal variants and the role of rare variants [65, 66]. Consequently, the plausible signals of selection here found are expected to be weak and difficult to be clearly identified. In this way, different novel method to detect selection are needed to address potential confounding effects caused by population history and structure and to establish definitive evidence of selection, mechanism of selection, and functional effects of the allelic variants under selection in complex traits.

One possible limitation of this survey is related to the ascertainment bias in the selection of marker. The fact that these results were obtained by genotyping SNPs selected for specific criteria (MAF higher than 0.05 and giving priority to markers not in LD in European populations), and not through direct sequencing, could affect the found patterns of allele frequency, LD and population differentiation [5]. It has been reported that the ascertainment bias introduced by many methods of SNP discovery may have a large effect on the estimation of LD and recombination, influencing the decay of LD with distance [67]. Several studies reported significant LD over distances longer than those predicted by standard models, whereas some data from short, intergenic regions showed less LD than would be expected [68]. Indeed, loci containing high frequency alleles tend to have deeper than average genealogies, providing more opportunity for recombination, and thereby less LD. Regions in which many sequences are used for ascertainment show more SNPs at low frequencies and a higher number of haplotypes are represented in the data. In order to improve this bias, all the variation present in the 1000 Genomes Project [11] for these genomic regions was used to perform the F_ST_, the LRH and the Tajima’s D test. The correspondence in the results across these two datasets with different marker coverage (367 SNPs from our genotyping dataset and all the genetic variation from the 1000 Genomes Project) allow us to consider the 9p21 genomic region as a potential candidate for positive and balancing selection, and the 10q11 for balancing selection.

In the absence of additional functional information, our results are compatible with a potential selective role of CAD in shaping the genetic diversity observed in current human populations, but demographic processes cannot be discarded.

## Supporting Information

S1 Dataset Individual genotypes of the analysed data. (XLSX)

S1 Fig Genomic coordinates, genes included and genotyped SNPs for the region 1p13. (TIF)

S2 Fig Genomic coordinates, genes included and genotyped SNPs for the region 1q41. (TIF)

S3 Fig Genomic coordinates, genes included and genotyped SNPs for the region 9p21. (TIF)

S4 Fig Genomic coordinates, genes included and genotyped SNPs for the region 10q11. (TIF)

S5 Fig LD blocks for the risk region 1p13 in 26 populations from Europe, Africa and Asia. (TIF)

S6 Fig LD blocks for the risk region 1q41 in 26 populations from Europe, Africa and Asia. (TIF)

S7 Fig LD blocks for the risk region 9p21 in 26 populations from Europe, Africa and Asia. (TIF)

S8 Fig LD blocks for the risk region 10q11 in 26 populations from Europe, Africa and Asia. (TIF)

S9 Fig LD blocks for the risk region 1p13 in which markers previously associated with CAD are located in 26 populations from Europe, Africa and Asia. Markers previously associated in Europe are highlighted in blue.
(TIF)

S10 Fig LD blocks for the risk region 1q41 in which markers previously associated with CAD are located in 26 populations from Europe, Africa and Asia. Markers previously associated in Europe are highlighted in blue.
(TIF)

S1 Table Geographic origin, population codification, sample size and geographic coordinates in decimal degrees for the population samples. (XLSX)

S2 Table Allele frequencies and heterozygozities (mean and standard deviation (SD)) per marker and per population. (XLSX)

S3 Table Reynolds’s genetic distances estimated among North European and Mediterranean populations based on the SNPs located in the 1p13, 1q41, 9p21, and 10q11 CAD risk regions. (XLSX)

S4 Table LD statistics (D’ and r^2^) for each pair of risk SNPs and for each population analysed. Risk SNPs in the same haplotype are highlighted in red. Risk SNPs in the same haplotype block in LD (D’ = 1) are highlighted in grey.
(XLSX)

S5 Table Haplotype block data for the 1p13 region based on the S9 Fig. CAD risk SNPs are highlighted in grey and their number corresponds to the risk markers in S9 Fig.
(XLSX)

S6 Table Haplotype block data for the 9p21 region based on the Fig 3. CAD risk SNPs are highlighted in grey and their number corresponds to the risk markers in Fig 3.
(XLSX)

S7 Table Haplotype block data for the 10q11 region based on the Fig 4. CAD risk SNPs are highlighted in grey and their number corresponds to the risk markers in Fig 4.
(XLSX)

S8 Table Detection of selection results for the four genomic regions analysed (1p13, 1q41, 9p21 and 10q11). Observed (Obs) F_ST_ with selection significance (highlighted P value<0.01) for the three continents (Africa, Asia and Europe) and for Europe and North Africa. Global cross-continental F_ST_ scores in the CEU, CHB, and YRI samples from the 1000 Genomes Selection Browser. Significant high and low FST values (extreme top 5% values of the distribution of F_ST_ values across the genome) are highlighted in grey. P values were calculated on the basis of higher rank scores in the genomic distribution. SPA scores for continental European, and North African allele gradients (highlighted in grey SPA scores above 99th percentile). Tajima’s D test results in the CEU, CHB, and YRI samples of the 1000 Genomes Project. Signals of potential balancing selection (P value≤0.05) are highlighted in grey. P-values were calculated on the basis of higher rank scores in the genomic distribution of Tajima’s D values.
(XLSX)

S9 Table Global cross-continental F_ST_ scores in the CEU, CHB, and YRI samples of the 1000 Genomes Project. Significant high and low F_ST_ values (above the top 5% extreme values of the distribution of F_ST_ values across de genome) are highlighted in grey. P values were calculated on the basis of higher rank scores in the genomic distribution. Significant F_ST_ values after applying correction for multiple testing are highlighted in red.
(XLSX)

S10 Table EHH averages in the CEU, CHB, and YRI samples of the 1000 Genomes Project. Significant high EHH values (above the top 5% extreme values of the distribution of EHH values across de genome) are highlighted in grey. The region containing the CAD risk marker rs1333042 is highlighted in grey. P values were calculated on the basis of higher rank scores in the genomic distribution.
(XLSX)

## References

[1] McPherson R, Pertsemlidis A, Kavaslar N, Stewart A, Roberts R, Cox DR, et al A common allele on chromosome 9 associated with coronary heart disease. Science. 2007;316: 1488–91. 10.1126/science.1142447 17478681 PMC2711874

[2] Helgadottir A, Thorleifsson G, Manolescu A, Gretarsdottir S, Blondal T, Jonasdottir A, et al A common variant on chromosome 9p21 affects the risk of myocardial infarction. Science. 2007;316: 1491–3. 10.1126/science.1142842 17478679

[3] Roberts R. Genetics of Coronary Artery Disease: An Update. Methodist Debakey Cardiovasc J. 2014;10: 7–12. 10.14797/mdcj-10-1-7 24932356 PMC4051327

[4] Roberts R, Stewart AFR. Genes and coronary artery disease: where are we? J Am Coll Cardiol. Elsevier Inc. 2012;60: 1715–21. 10.1016/j.jacc.2011.12.062 23040572

[5] Nielsen R, Hellmann I, Hubisz M, Bustamante C, Clark AG. Recent and ongoing selection in the human genome. Nat Rev Genet. 2007;8: 857–68. 10.1038/nrg2187 17943193 PMC2933187

[6] Corona E, Dudley JT, Butte AJ. Extreme evolutionary disparities seen in positive selection across seven complex diseases. PLoS One. 2010;5: e12236 10.1371/journal.pone.0012236 20808933 PMC2923198

[7] Ding K, Kullo IJ. Geographic differences in allele frequencies of susceptibility SNPs for cardiovascular disease. BMC Med Genet. BioMed Central Ltd. 2011;12: 55 10.1186/1471-2350-12-55 21507254 PMC3103418

[8] Hancock AM, Witonsky DB, Alkorta-Aranburu G, Beall CM, Gebremedhin A, Sukernik R, et al Adaptations to climate-mediated selective pressures in humans. PLoS Genet. 2011;7: e1001375 10.1371/journal.pgen.1001375 21533023 PMC3080864

[9] Soranzo N, Spector TD, Mangino M, Kühnel B, Rendon A, Teumer A, et al A genome-wide meta-analysis identifies 22 loci associated with eight hematological parameters in the HaemGen consortium. Nat Genet. Nature Publishing Group. 2009;41: 1182–90. 10.1038/ng.467 PMC3108459 19820697

[10] Zanetti D, Via M, Carreras-Torres R, Esteban E, Chaabani H, Anaibar F, et al (2015) Analysis of genomic regions associated with Coronary Artery Disease reveals continental-specific risk SNPs in North African populations. Submitted to Journal of Epidemiology. 10.2188/jea.JE20150034 PMC4848325 26780859

[11] Abecasis GR, Auton A, Brooks LD, DePristo M a, Durbin RM, Handsaker RE, et al An integrated map of genetic variation from 1,092 human genomes. Nature. 2012;491: 56–65. 10.1038/nature11632 23128226 PMC3498066

[12] Broadbent HM, Peden JF, Lorkowski S, Goel A, Ongen H, Green F, et al Susceptibility to coronary artery disease and diabetes is encoded by distinct, tightly linked SNPs in the ANRIL locus on chromosome 9p. Hum Mol Genet. 2008;17: 806–14. 10.1093/hmg/ddm352 18048406

[13] Lee J-Y, Lee B-S, Shin D-J, Woo Park K, Shin Y-A, Joong Kim K, et al A genome-wide association study of a coronary artery disease risk variant. J Hum Genet. Nature Publishing Group. 2013;58: 120–6. 10.1038/jhg.2012.124 23364394

[14] Li X, Huang Y, Yin D, Wang D, Xu C, Wang F, et al Meta-analysis identifies robust association between SNP rs17465637 in MIA3 on chromosome 1q41 and coronary artery disease. Atherosclerosis. Elsevier Ireland Ltd. 2013;231: 136–40. 10.1016/j.atherosclerosis.2013.08.031 24125424

[15] Esparragón FR, Companioni O, Bello MG, Ríos NB, Pérez JCR. Replication of relevant SNPs associated with cardiovascular disease susceptibility obtained from GWAs in a case-control study in a Canarian population. Dis Markers. 2012;32: 231–9. 10.3233/DMA-2011-0879 22430189 PMC3826386

[16] Purcell S, Neale B, Todd-Brown K, Thomas L, Ferreira M a R, Bender D, et al PLINK: a tool set for whole-genome association and population-based linkage analyses. Am J Hum Genet. 2007;81: 559–75. 10.1086/519795 17701901 PMC1950838

[17] R Core Team (2013) R: A language and environment for statistical computing R Foundation for Statistical Computing, Vienna, Austria ISBN 3-900051-07-0, URL http://www.R-project.org/.

[18] Excoffier L, Lischer HEL. Arlequin suite ver 3.5: a new series of programs to perform population genetics analyses under Linux and Windows. Mol Ecol Resour. 2010;10: 564–7. 10.1111/j.1755-0998.2010.02847.x 21565059

[19] Jombart T. adegenet: a R package for the multivariate analysis of genetic markers. Bioinformatics. 2008;24: 1403–5. 10.1093/bioinformatics/btn129 18397895

[20] Josse J. FactoMineR : An R Package for Multivariate Analysis. 2008;25: 1–18.

[21] Gabriel SB, Schaffner SF, Nguyen H, Moore JM, Roy J, Blumenstiel B, et al The structure of haplotype blocks in the human genome. Science 2002;296: 2225–2229. 10.1126/science.1069424 12029063

[22] Gu S, Pakstis AJ, Kidd KK. HAPLOT: a graphical comparison of haplotype blocks, tagSNP sets and SNP variation for multiple populations. Bioinformatics. 2005;21: 3938–9. 10.1093/bioinformatics/bti649 16131520

[23] Prins BP, Lagou V, Asselbergs FW, Snieder H, Fu J. Genetics of coronary artery disease: genome-wide association studies and beyond. Atherosclerosis. Elsevier Ltd. 2012;225: 1–10. 10.1016/j.atherosclerosis.2012.05.015 22698794

[24] Dichgans M, Malik R, König IR, Rosand J, Clarke R, Gretarsdottir S, et al Shared genetic susceptibility to ischemic stroke and coronary artery disease: a genome-wide analysis of common variants. Stroke. 2014;45: 24–36. 10.1161/STROKEAHA.113.002707 24262325 PMC4112102

[25] Lu X, Wang L, Chen S, He L, Yang X, Shi Y, et al Genome-wide association study in Han Chinese identifies four new susceptibility loci for coronary artery disease. Nat Genet. 2012;44: 890–4. 10.1038/ng.2337 22751097 PMC3927410

[26] Ong RT-H, Teo Y-Y. varLD: a program for quantifying variation in linkage disequilibrium patterns between populations. Bioinformatics. 2010;26: 1269–70. 10.1093/bioinformatics/btq125 20308177

[27] Barrett JC, Fry B, Maller J, Daly MJ. Haploview: analysis and visualization of LD and haplotype maps. Bioinformatics. 2005;21: 263–5. 10.1093/bioinformatics/bth457 15297300

[28] Excoffier L, Hofer T, Foll M. Detecting loci under selection in a hierarchically structured population. Heredity (Edinb). Nature Publishing Group. 2009;103: 285–98. 10.1038/hdy.2009.74 19623208

[29] Yang W-Y, Novembre J, Eskin E, Halperin E. A model-based approach for analysis of spatial structure in genetic data. Nat Genet. Nature Publishing Group. 2012;44: 725–31. 10.1038/ng.2285 22610118 PMC3592563

[30] Relethford JH. Geostatistics and spatial analysis in biological anthropology. Am J Phys Anthropol. 2008;136: 1–10. 10.1002/ajpa.20789 18257009

[31] Tajima F. Statistical Method for Testing the Neutral Mutation Hypothesis by DNA Polymorphism. 1989;595: 585–595 10.1093/genetics/123.3.585 PMC1203831 2513255

[32] Pybus M, Dall’Olio GM, Luisi P, Uzkudun M, Carreño-Torres A, Pavlidis P, et al 1000 Genomes Selection Browser 1.0: A genome browser dedicated to signatures of natural selection in modern humans. Nucleic Acids Res. 2014;42: 903–909. 10.1093/nar/gkt1188 PMC3965045 24275494

[33] Benjamini Y, Hochberg Y. Controlling the False Discovery Rate: A practical and powerful Approach to Multiple Testing. Journal of the Royal Statistical Society. Series B (Methodological). 1995;pp:289–300

[34] Lettre G, Palmer CD, Young T, Ejebe KG, Allayee H, Benjamin EJ, et al Genome-wide association study of coronary heart disease and its risk factors in 8,090 African Americans: the NHLBI CARe Project. PLoS Genet. 2011;7: e1001300 10.1371/journal.pgen.1001300 21347282 PMC3037413

[35] Larson MG, Atwood LD, Benjamin EJ, Cupples LA, D’Agostino RB, Fox CS, et al Framingham Heart Study 100K project: genome-wide associations for cardiovascular disease outcomes. BMC Med Genet. 2007;8 Suppl 1: S5 10.1186/1471-2350-8-S1-S5 17903304 PMC1995607

[36] Heckman MG, Soto-ortolaza AI, Diehl NN, Brott TG, Wszolek ZK, Meschia JF, et al Genetic variants associated with myocardial infarction in the PSMA6 gene and Chr9p21 are also associated with ischaemic stroke. Eur J Neurol. 2014;20: 300–308. 10.1111/j.1468-1331.2012.03846.x PMC3711397 22882272

[37] Holdt LM, Teupser D. Recent studies of the human chromosome 9p21 locus, which is associated withatherosclerosis in human populations. Arterioscler Thromb Vasc Biol. 2012;32(2):196–206. 10.1161/ATVBAHA.111.232678 22258902

[38] Li JZ, Absher DM, Tang H, Southwick AM, Casto AM, Ramachandran S, et al Worldwide human relationships inferred from genome-wide patterns of variation. Science. 2008;319(5866):1100–4. 10.1126/science.1153717 18292342

[39] Zanetti D, Sadiq M, Carreras-torres R, Khabour O, Alkaraki A, Esteban E, et al Human Diversity in Jordan: polymorphic Alu insertions in general Jordanian and Bedouin groups. Hum Biol. 2014;86(2):131–8 25397703 10.3378/027.086.0201

[40] Novembre J, Johnson T, Bryc K, Kutalik Z, Boyko AR, Auton A, et al Genes mirror geography within Europe. Nature. 2008;456: 98–101. 10.1038/nature07331 18758442 PMC2735096

[41] Haber M, Gauguier D, Youhanna S, Patterson N, Moorjani P, Botigué LR, et al Genome-wide diversity in the levant reveals recent structuring by culture. PLoS Genet. 2013;9: e1003316 10.1371/journal.pgen.1003316 23468648 PMC3585000

[42] Athanasiadis G, Moral P. Spatial principal component analysis points at global genetic structure in the Western Mediterranean. J Hum Genet. Nature Publishing Group. 2013;58: 762–5. 10.1038/jhg.2013.94 24005895

[43] Lao O, Lu TT, Nothnagel M, Junge O, Freitag-Wolf S, Caliebe A, et al Correlation between Genetic and Geographic Structure in Europe. Curr Biol. 2008;18: 1241–1248. 10.1016/j.cub.2008.07.049 18691889

[44] Disotell TR. Archaic human genomics. Am J Phys Anthropol. 2012;149 Suppl: 24–39. 10.1002/ajpa.22159 23124308

[45] Trompet S, de Craen AJM, Postmus I, Ford I, Sattar N, Caslake M, et al Replication of LDL GWAs hits in PROSPER/PHASE as validation for future (pharmaco)genetic analyses. BMC Med Genet. BioMed Central Ltd. 2011;12: 131 10.1186/1471-2350-12-131 21977987 PMC3207930

[46] Sanna S, Li B, Mulas A, Sidore C, Kang HM, Jackson AU, et al Fine mapping of five loci associated with low-density lipoprotein cholesterol detects variants that double the explained heritability. PLoS Genet. 2011;7 10.1371/journal.pgen.1002198 PMC3145627 21829380

[47] Munir MS, Wang Z, Alahdab F, Steffen MW, Erwin PJ, Kullo IJ, et al The association of 9p21-3 locus with coronary atherosclerosis: a systematic review and meta-analysis. BMC Med Genet. 2014;15: 66 10.1186/1471-2350-15-66 24906238 PMC4074865

[48] Bersaglieri T, Sabeti PC, Patterson N, Vanderploeg T, Schaffner SF, Drake JA, et al Genetic signatures of strong recent positive selection at the lactase gene. Am J Hum Genet. 2004;74: 1111–1120. 10.1086/421051 15114531 PMC1182075

[49] Pickrell JK, Coop G, Novembre J, Kudaravalli S, Li JZ, Absher D, et al Signals of recent positive selection in a worldwide sample of human populations. Genome Res. 2009;19(5):826–837. 10.1101/gr.087577.108 19307593 PMC2675971

[50] Tunstall-Pedoe H, Kuulasmaa K, Mähönen M, Tolonen H, Ruokokoski E, Amouyel P. Contribution of trends in survival and coronary-event rates to changes in coronary heart disease mortality: 10-year results from 37 WHO MONICA Project populations. Lancet. 1999; 353(9164):1547–57. 10.1016/S0140-6736(99)04021-0 10334252

[51] Stengård J H, Weiss KM, Sing CF. An ecological study of association between coronary heart disease mortality rates in men and the relative frequencies of common allelic variations in the gene coding for apolipoprotein E. Hum Genet. 1998;103, 234–241. 10.1007/s004390050811 9760210

[52] Carreras-Torres R, Kundu S, Zanetti D, Esteban E, Via M, Moral P. Genetic Risk Score of NOS Gene Variants Associated with Myocardial Infarction Correlates with Coronary Incidence across Europe. PLoS ONE. 2014;9(5), e96504 10.1371/journal.pone.0096504 24806096 PMC4013019

[53] Lao O, Dupanloup I, Barbujani G, Bertranpetit J, Calafell F. The mediterranean paradox for susceptibility factors in coronary heart disease extends to genetics. Ann Hum Genet. 2008;72: 48–56. 10.1111/j.1469-1809.2007.00387.x 17683517

[54] Mcevoy BP, Montgomery GW, Mcrae AF, Ripatti S, Perola M, Spector TD, et al Geographical structure and differential natural selection among North European populations Geographical structure and differential natural selection among North European populations. Genome Res. 2009;19(5):804–814. 10.1101/gr.083394.108 19265028 PMC2675969

[55] Casto AM, Feldman MW. Genome-wide association study SNPs in the human genome diversity project populations: does selection affect unlinked SNPs with shared trait associations? PLoS Genet. 2011;7: e1001266 10.1371/journal.pgen.1001266 21253569 PMC3017115

[56] Hancock AM, Witonsky DB, Alkorta-Aranburu G, Beall CM, Gebremedhin A, Sukernik R, et al Adaptations to climate-mediated selective pressures in humans. PLoS Genet. 2011; 7(4). 10.1371/journal.pgen.1001375 PMC3080864 21533023

[57] Ding K, Kullo IJ. Evolutionary genetics of coronary heart disease. Circulation. 2009;119: 459–467. 10.1161/CIRCULATIONAHA.108.809970 19171868

[58] Wang ET, Kodama G, Baldi P, Moyzis RK. Global landscape of recent inferred Darwinian selection for Homo sapiens. Proc Natl Acad Sci U S A. 2006;103: 135–140. 10.1073/pnas.0509691102 16371466 PMC1317879

[59] Li W-Q, Pfeiffer RM, Hyland PL, Shi J, Gu F, Wang Z, et al Genetic polymorphisms in the 9p21 region associated with risk of multiple cancers. Carcinogenesis. 2014;35: 2698–2705. 10.1093/carcin/bgu203 25239644 PMC4247519

[60] Burdon KP, Macgregor S, Hewitt AW, Sharma S, Chidlow G, Mills RA, et al Genome-wide association study identifies susceptibility loci for open angle glaucoma at TMCO1 and CDKN2B-AS1. Nat Genet. Nature Publishing Group. 2011;43: 574–8. 10.1038/ng.824 21532571

[61] Helgadottir A, Thorleifsson G, Magnusson KP, Grétarsdottir S, Steinthorsdottir V, Manolescu A, et al The same sequence variant on 9p21 associates with myocardial infarction, abdominal aortic aneurysm and intracranial aneurysm. Nat Genet. 2008;40: 217–24. 10.1038/ng.72 18176561

[62] Emanuele E, Lista S, Ghidoni R, Binetti G, Cereda C, Benussi L, et al Chromosome 9p21.3 genotype is associated with vascular dementia and Alzheimer’s disease. Neurobiol Aging. Elsevier Inc. 2011;32: 1231–5. 10.1016/j.neurobiolaging.2009.07.003 19664850

[63] Teshima KM, Coop G, Przeworski M. How reliable are empirical genomic scans for selective sweeps? Genome Res. 2006;16: 702–712. 10.1101/gr.5105206 16687733 PMC1473181

[64] Pritchard JK, Pickrell JK, Coop G. The genetics of human adaptation: hard sweeps, soft sweeps, and polygenic adaptation. Curr Biol. 2010;20(4):R208–15. 10.1016/j.cub.2009.11.055 20178769 PMC2994553

[65] Gibson G. Rare and common variants: twenty arguments. Nat Rev Genet. 2012;13:135–145. 10.1038/nrg3118 22251874 PMC4408201

[66] Bustamante CD, Burchard EG, De la Vega FM. Genomics for the world. Nature. 2011;475:163–165. 10.1038/475163a 21753830 PMC3708540

[67] Nielsen R, Signorovitch J. Correcting for ascertainment biases when analyzing SNP data: Applications to the estimation of linkage disequilibrium. Theor Popul Biol. 2003;63:245–255. 10.1016/S0040-5809(03)00005-4 12689795

[68] Pritchard JK, Przeworski M. Linkage disequilibrium in humans: models and data. Am J Hum Genet. 2001;69:1–14. 10.1086/321275 11410837 PMC1226024

