# Supplementary figures and images for: Potential Signals of Natural Selection in the Top Risk Loci for Coronary Artery Disease: 9p21 and 10q11
Source: PLoS One. 2015 Aug 7;10(8):e0134840. doi: 10.1371/journal.pone.0134840 (PMC4529309; doi:10.1371/journal.pone.0134840)

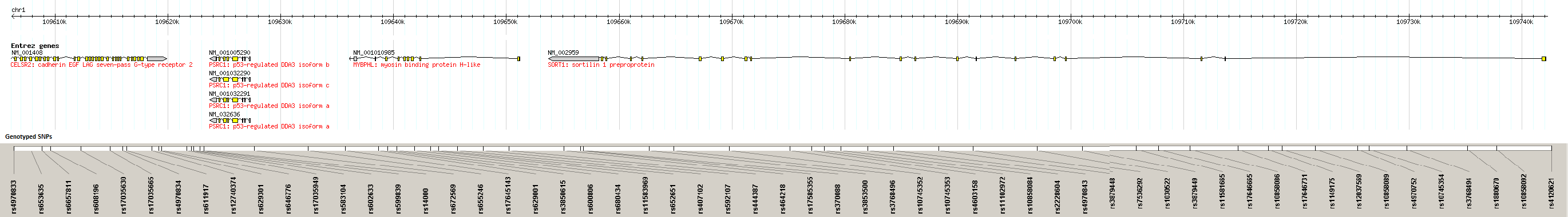

Supplement: S1 Fig — (TIF) [file pone.0134840.s002.tif]

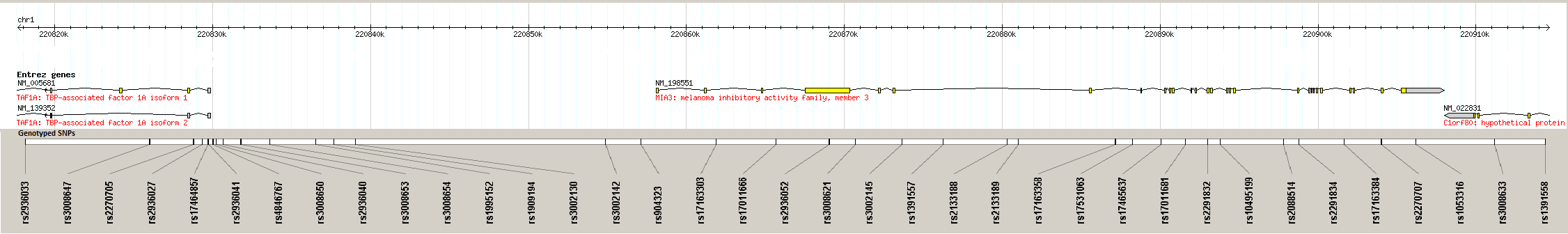

Supplement: S2 Fig — (TIF) [file pone.0134840.s003.tif]

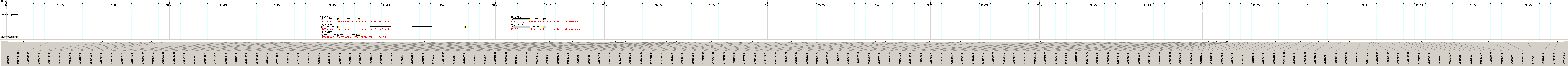

Supplement: S3 Fig — (TIF) [file pone.0134840.s004.tif]

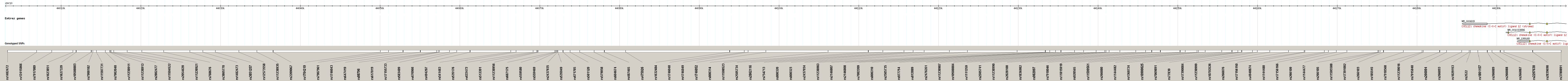

Supplement: S4 Fig — (TIF) [file pone.0134840.s005.tif]

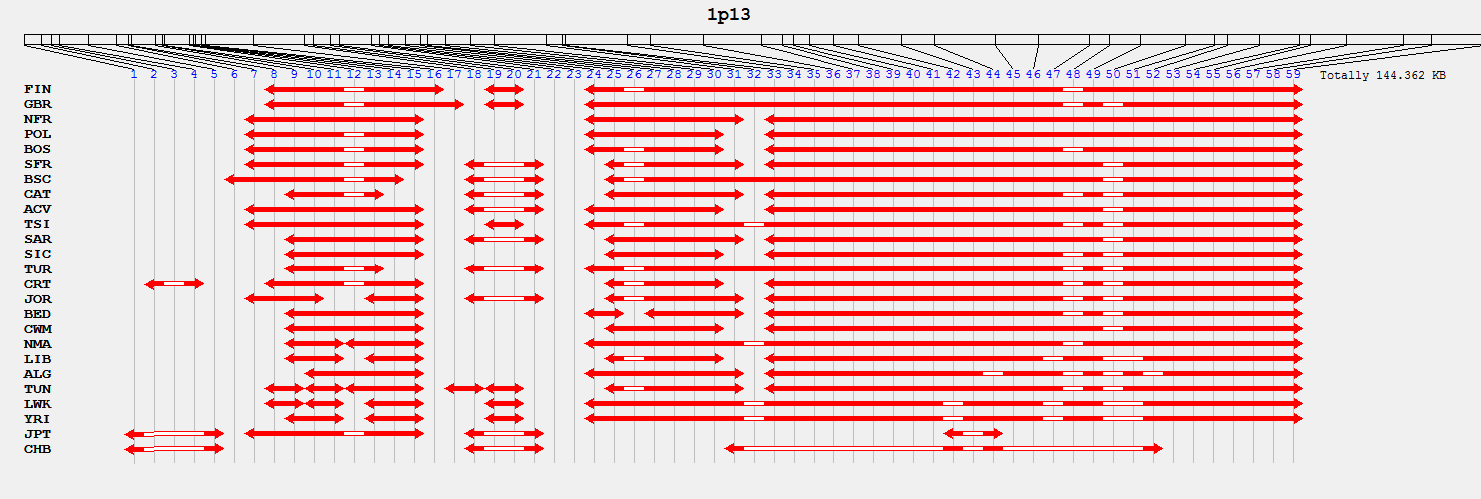

Supplement: S5 Fig — (TIF) [file pone.0134840.s006.tif]

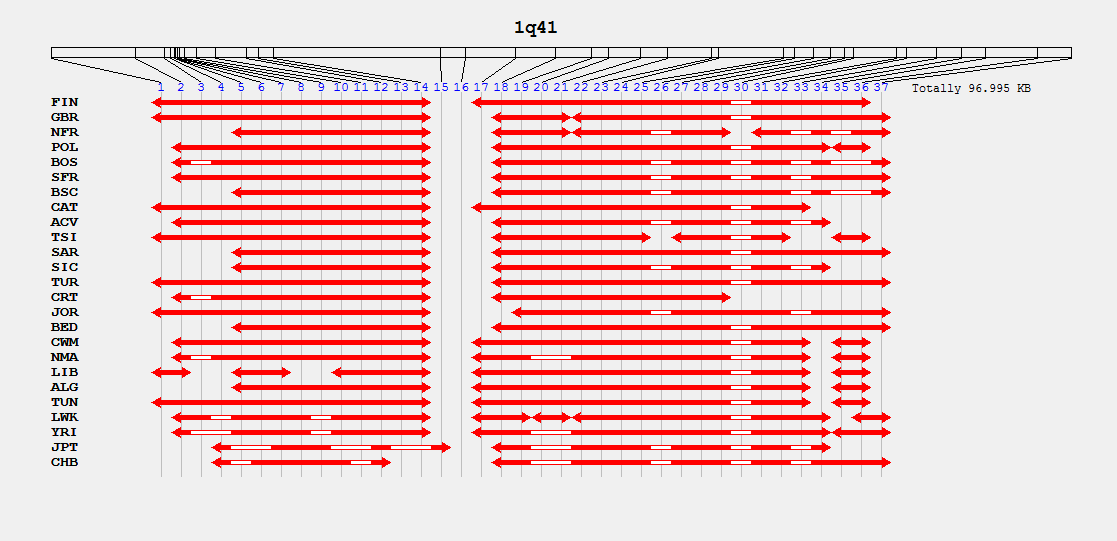

Supplement: S6 Fig — (TIF) [file pone.0134840.s007.tif]

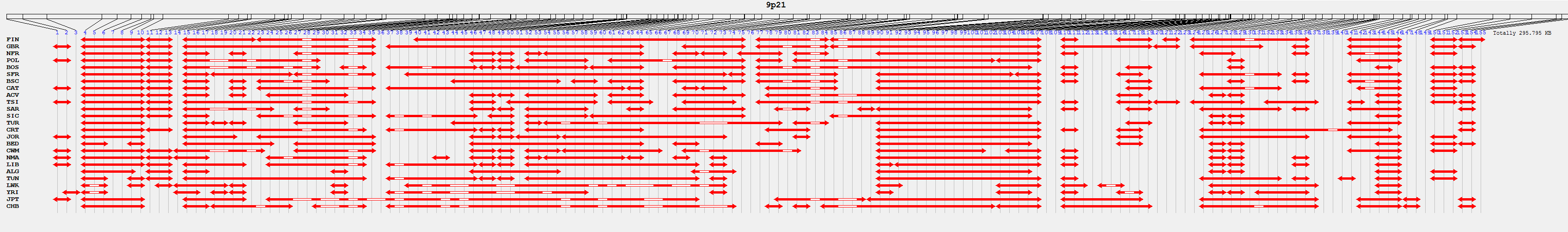

Supplement: S7 Fig — (TIF) [file pone.0134840.s008.tif]

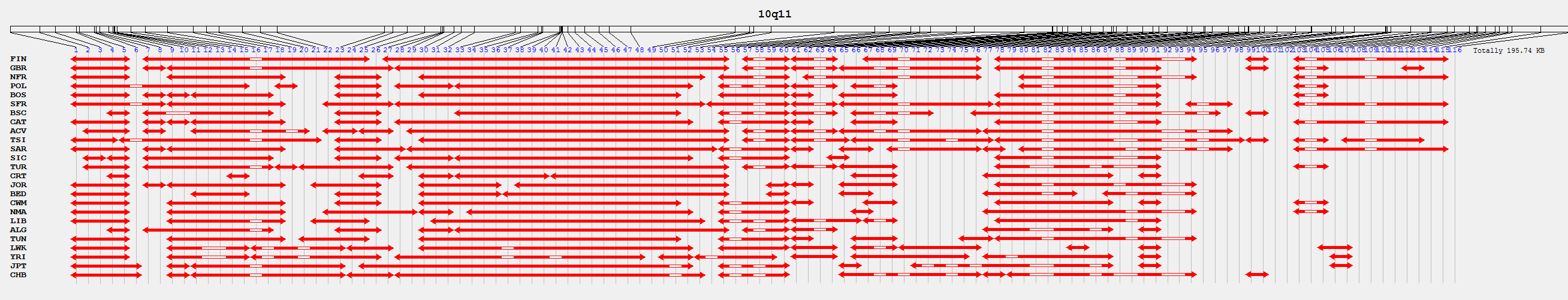

Supplement: S8 Fig — (TIF) [file pone.0134840.s009.tif]

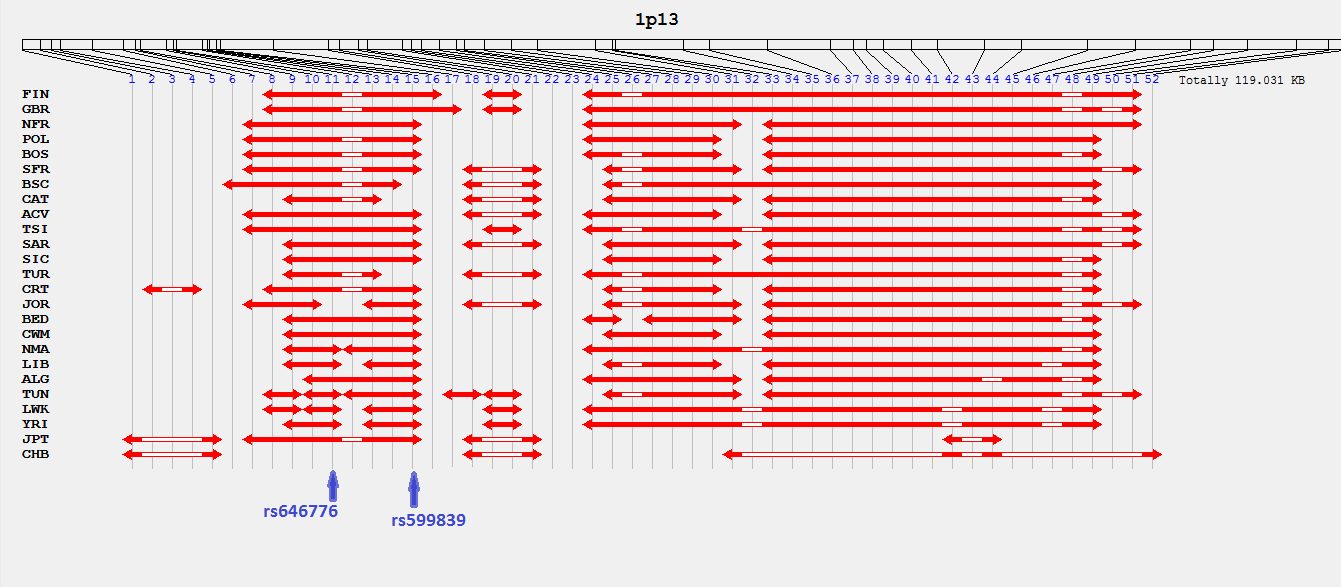

Supplement: S9 Fig — Markers previously associated in Europe are highlighted in blue. (TIF) [file pone.0134840.s010.tif]

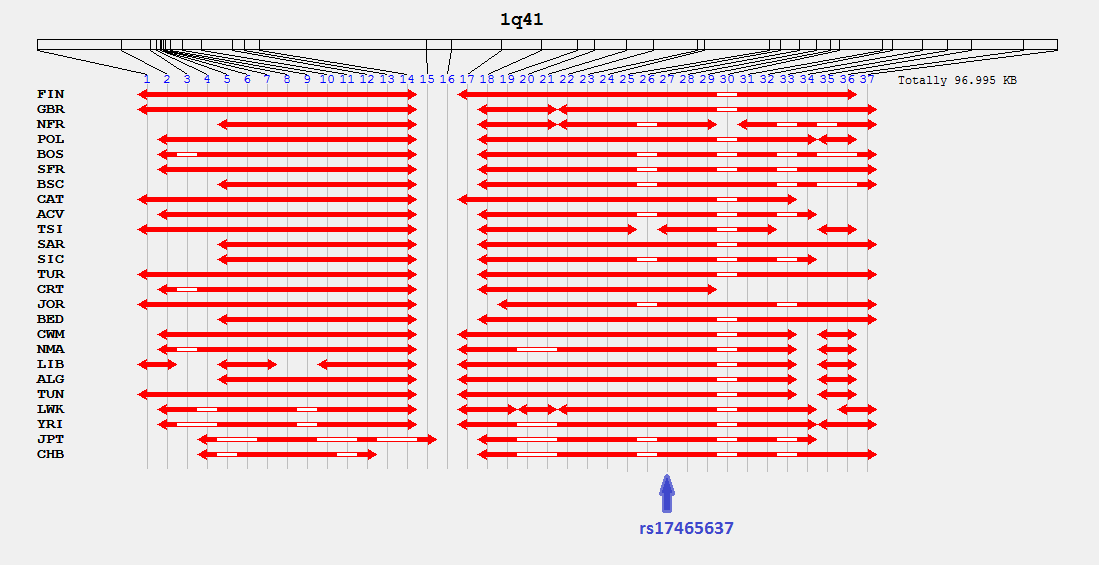

Supplement: S10 Fig — Markers previously associated in Europe are highlighted in blue. (TIF) [file pone.0134840.s011.tif]
